# Intra-articular corticosteroid injections for osteoarthritis: A qualitative study of patients’ and clinicians’ experiences

**DOI:** 10.1371/journal.pone.0311668

**Published:** 2024-10-23

**Authors:** Andrew J. Moore, Cecily K. Palmer, Karen L. Barker, Rachael Gooberman-Hill, Andy Judge, Vikki Wylde, Michael R. Whitehouse

**Affiliations:** 1 University of Bristol, Bristol Medical School, Bristol, United Kingdom; 2 Oxford University Hospitals NHS Trust, Oxford, United Kingdom; University of Naples Federico II: Universita degli Studi di Napoli Federico II, ITALY

## Abstract

**Background:**

Osteoarthritis is a leading cause of joint pain and disability. Intra-articular corticosteroid injections (IACs) are often used in primary care once other recommended treatments have failed. Evidence shows that IACs provide short-term relief of osteoarthritis symptoms, yet little is known about patients’ and primary care clinicians’ experiences and beliefs about their use. We explored patients’ and primary care clinicians’ views about IACs, including the benefits, disadvantages, perceived risks of treatment, when they are used, and factors that affect decision-making.

**Methods:**

We conducted individual interviews with patients and primary care clinicians and used inductive thematic analysis to investigate their views and experiences of intra-articular corticosteroid injections for osteoarthritis (IACs).

**Findings:**

We interviewed 38 patients and 19 primary care clinicians. We identified 6 patient themes: variation in access; awareness of IACs; views of risk and trust; effectiveness of IACs; variation in onset and effect duration; and an alternative to undesirable treatments. In the interviews with clinicians, we identified an overarching theme of caution and competence, which included eight subthemes: confidence and (dis)comfort with practical procedures; risk of adverse outcomes; training; uncertainty about evidence and guidelines; technical uncertainties; IACs use on the osteoarthritis pathway; perceived benefits and impacts of IACs; and the possibility of placebo.

**Conclusion:**

Patients and clinicians valued IACs’ potential to relieve symptoms and improve quality of life. Variability in patients’ access to treatment appears related to clinicians’ confidence in delivering injections and their concerns about the evidence base. Variation in dose frequency and timing reflect clinicians’ uncertainty about current guidance. Despite variation in effectiveness patients preferred IACs to other forms of pain medication and to delay or avoid surgery. IACs were mostly used as an adjunct treatment before surgery was offered. These findings can inform further research into the effectiveness of IACs and improvements in information and guidance.

## Introduction

Osteoarthritis is a leading cause of pain and disability worldwide [1]. In the United Kingdom (UK) approximately 10% of adults have the condition. It has a significant impact on individual’s health and wellbeing, affecting them physically, emotionally and financially. The personal impact of osteoarthritis includes limitation of activities of daily living and participation in valued activities, sleep disturbance, reduced ability to work, lowered self-worth, frustration, depression and anxiety [2, 3]. Approximately half of all those with osteoarthritis do not seek help until their pain is unbearable, and two thirds report persistent pain and functional restrictions despite use of medications [4].

Despite the impact of osteoarthritis on patients, society and the National Health Service (NHS), there are few therapeutic options for osteoarthritis [5–7]. Some non-pharmacological treatments such as physiotherapy and exercise show beneficial effects on pain and function [8, 9] but many non-pharmacologic treatments suffer from poor adherence [10–13]. Pharmacologic treatments such as non-steroidal anti-inflammatory drugs (NSAIDs) are associated with serious side-effects [14] or lack strong evidence of benefit [5, 7]. People whose symptoms do not respond to conservative management are frequently referred for joint replacement surgery.

Intra-articular corticosteroid injections (IACs) are used to deliver high doses of synthetic corticosteroids into a specific joint. Current guidance recommends their use when other pharmacological treatments are ineffective or unsuitable, or to support therapeutic exercise [7]. Although there is considerable evidence that they provide short-term relief from symptoms for up to 3 months, there is a lack of evidence of longer-term efficacy and safety [15–18]. For example, there is conflicting evidence over whether recurrent use of IACs is associated with increased progression of cartilage loss in osteoarthritis [19–22], or an increase in the risk of infection after joint arthroplasty [23–25]. With rising prevalence of osteoarthritis, it is likely that IACs become used more frequently and this highlights an urgent need for robust evidence about the long-term benefits and risks associated with the use of IACs for osteoarthritis. Evidence about patients’ and clinicians’ experiences, beliefs and motivations for use of IACs, alongside information about factors that affect their decision-making can be used to inform recommendations for practice and policy.

We aimed to explore patients’ experiences and views about receipt of IACs for osteoarthritis and the views and experiences of primary care clinicians who administer IACs for osteoarthritis.

## Methods

### Study design and setting

We conducted one-to-one, semi-structured telephone/videocall interviews with patients and primary care clinicians to investigate their views and experiences of IACs. Interviews were conducted between 29^th^ January 2021 and 15^th^ December 2021. A purposive sampling strategy was used to identify and recruit patients and clinicians from across a range of primary care practices in the Southwest of England. The study received ethical approval in July 2020 (REC ref. 20/EM/0185).

### Sampling and recruitment

A sample of 40 patients and 30 clinicians was planned as an approximation expected to yield adequate ‘information power’ to address the research questions [26]. As there is substantial variation in the sociodemographics of the population, and rural/urban practice setting across Southwest England the sample size was designed to provide an appropriate cross-section of the wider NHS while achieving sufficient depth.

Eligible participants included adults aged 45 years or more who had received IACs within a primary care setting, within the previous three years, including those who received surgical intervention, and those who had osteoarthritis but had not received IACs. Clinicians (General Practitioners and First Contact physiotherapy practitioners) from across Southwest England including those who administered IACs for osteoarthritis in the preceding 3 years and those who had not were also invited. Exclusion criteria were any individual who lacked capacity to provide informed consent, or who could not converse fluently in English as we had no resource for an interpreter to remove any language barriers.

Primary care practices screened patient information to identify eligible patients and then posted out information packs that described the purpose and aims of the study. We used a purposive maximum variation sampling approach [27] to ensure a diverse range of views and experiences within the sample, asking practices to diversify the sample by sex, age groups (45–59, 60–74 and 75+), ethnicity (White, Black, Asian and Minority ethnic groups), and joint injected (Knee, Hip, Shoulder, Elbow, Thumb or Wrist).

### Data collection

Before interview, participants provided informed consent either by electronic eConsent forms or verbal consent in keeping with the proportionate approach recommended by the Health Research Authority for low-risk non-interventional studies. Verbal consent was audio-recorded at the beginning of each interview, and a copy of a verbal consent form signed by the researcher was sent to participants for their records afterwards.

All interviews were conducted by the lead author AJM (male, PhD) who is an academic researcher and methodologist with over 14-years’ experience in conducting qualitative research on osteoarthritis pain and management. AJM had no relationship to the participants before this study. Interviews took place via telephone or Microsoft Teams. Topic guides were developed in collaboration with clinical team members and Patient and Public Involvement and Engagement (PPIE) representatives (See S1 & S2 Appendices) with questions framed around the broad aims of the study. PPIE group representatives suggested adding several key questions around access to IACs; how many GPs in a practice give injections; how many injections patients are told they can have, and how this might affect timing of surgery; and how long patients had to wait to get an injection. These questions were added to the patient and clinician topic guides. Topic guides were then piloted in the first two interviews. No alterations were needed to either topic guide. Data collection stopped when it was felt that there was sufficient information power within the sample, to meet the broad aims of the study, supported by a purposive sample and strong interview dialogue [26].

### Data analysis

Interview audio-recordings were transcribed, anonymised and uploaded to NVivo data management software [28]. Transcripts were analysed using a flexible, inductive approach to analysis to identify themes and sub-themes [29–31]. Coding of the patient and clinician interviews was concurrent. The transcripts were initially read alongside a review of the audio recording, before inductive coding was undertaken on a line-by-line basis. Data were analysed separately to enable comparison. To ensure rigour a second researcher independently analysed 25% of the transcripts (CP, female, PhD, experienced qualitative health services researcher) with both researchers meeting regularly to discuss each other’s suppositions and the developing codes and themes and interpretation of the findings. Differences in codes were discussed and either a new code was established, or a single name was chosen for consistency once it was clear that different codes shared a similar meaning. There were no notable disagreements about the findings. The analysis focused on the experience and perceptions of participants’ use of IACs, and as similarities and relationships between groups of codes were identified, they were aggregated into categories. The coded data were then reviewed, and these categories further refined into more conceptual themes that demonstrate meaning across the data set.

### Reflexivity

Throughout the study a reflexive approach was maintained. AJM kept post-interview field notes to record first impressions, thoughts or hypotheses, and any recurring themes [32]. AJM also met regularly with MRW (professor and orthopaedic consultant) to discuss and reflect on the analysis and developing interpretation.

## Results

**Fig 1:**
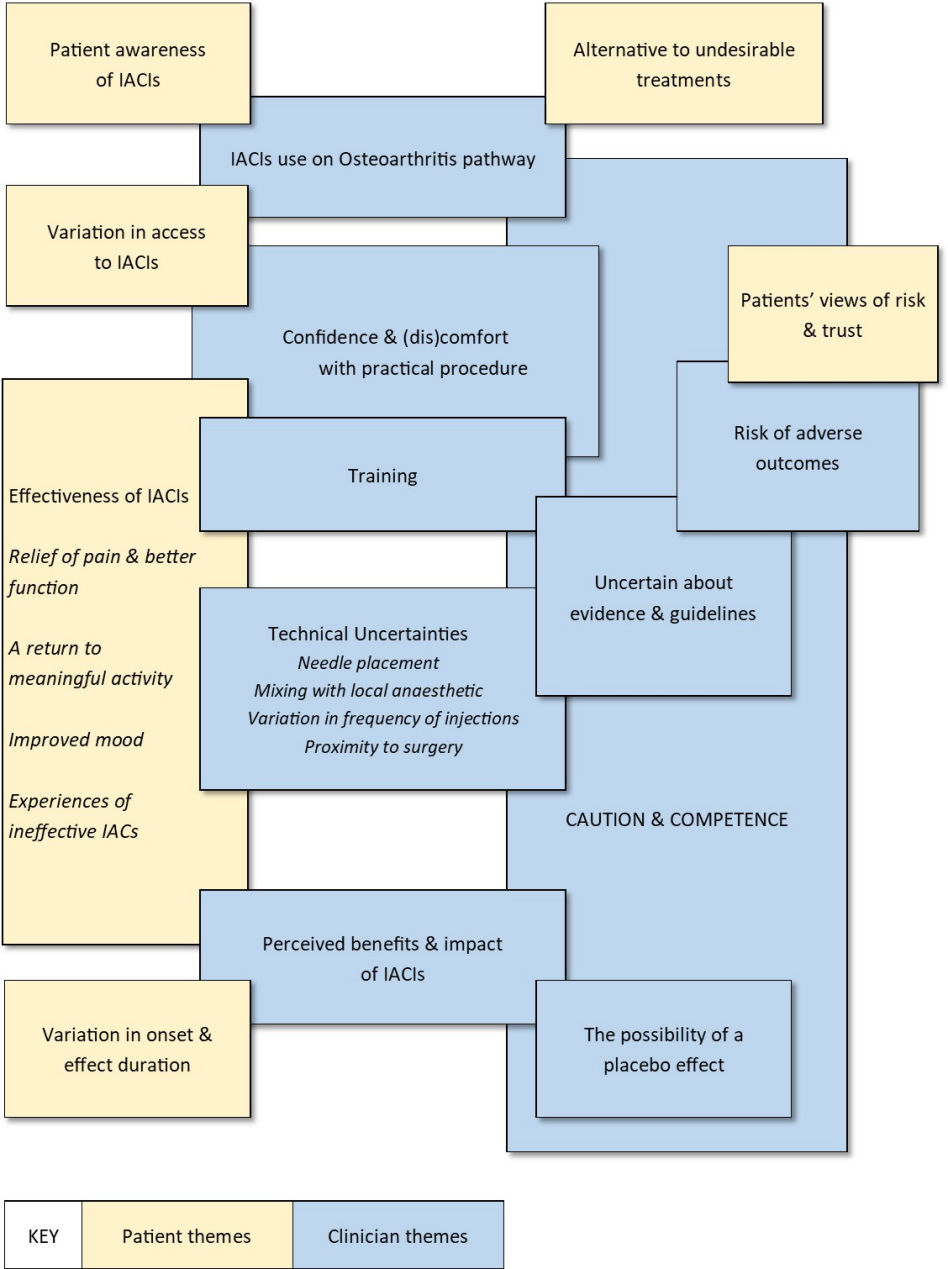
Thematic map of patient and clinician themes showing areas of overlap.

We present the results of our analysis in two parts, first describing the sample characteristics and themes based on patient accounts, before then describing those of the clinicians. We have used anonymised exemplary quotes to illustrate meaning within each theme. These can be seen in the thematic map in Fig 1 which illustrates the patient themes (yellow), clinician themes (blue) and areas of overlap.

### Patient participant characteristics

Sixteen male and 22 female patients participated, aged from 49 to 88 years (average 68.5 years) from six primary care practices. Twenty-eight had received one or more injections in their knee, although other joints injected included the shoulder, thumb, hip, hand, toe, foot and wrist. Five patients had not received IACs for osteoarthritis (see Table 1). Only one patient participant identified as “mixed race” while all others identified as white. Interviews lasted an average of 37 minutes (range: 21–80 minutes).

**Table 1:** Patient participant characteristics.

Participant ID	Sex (Male / Female)	Ethnicity (White, Black, Asian and Minority ethnic groups)	Age range at interview (years)	Joint(s) injected (number of times)	Single (x1) IACs in one or more joint	Multiple (x2 or more) IACs in one joint	Multiple (x2 or more) IACs in multiple joints
H1Pa	M	Mixed	60–74	Knee (x2)		✓	
H2pa	F	White	60–74	Knee (x3)		✓	
H3pa	M	White	45–59	Knee (x2)		✓	
H4pa	F	White	60–74	Both knees (x1)	✓		
E1pa	M	White	75+	None	-	-	-
E2pa	M	White	60–74	None	-	-	-
E3pa	M	White	60–74	Shoulder (x1) Both knees more than twice			✓
E4pa	F	White	75+	Wrist (x2) Thumb (x1)			✓
E5pa	F	White	60–74	Knee (x4)		✓	
E6pa	F	White	75+	None	-	-	-
E7pa	F	White	45–59	None	-	-	-
F1pa	M	White	60–74	Both shoulders injected (x4 in total)			✓
F3pa	M	White	45–59	Knee (x4)		✓	
F4pa	M	White	60–74	Both knees injected (more than twice)			✓
F6pa	F	White	60–74	Hands and knees injected alternate months (x12)			✓
F7pa	F	White	45–59	Both knees (more than twice)			✓
D2pa	F	White	75+	Knee (x1)	✓		
D3pa	F	White	60–74	Knee (x2)		✓	
D4pa	F	White	60–74	Both knees (more than twice)			✓
D5pa	M	White	60–74	Knee (x6) Hip (x1)			✓
D6pa	M	White	60–74	Shoulder (x1)	✓		
D7pa	F	White	60–74	Thumb (x1) Shoulder (x1) Knee (x1)	✓		
D8pa	F	White	45–59	Shoulder (20+) Knee (20+)			✓
B1pa	F	White	75+	Knee (x2)		✓	
B2pa	F	White	45–59	Knee (x1)	✓		
B3pa	M	White	60–74	Thumbs (x1), (x2)			✓
B4pa	M	White	75+	Both knees (x2)			✓
B5pa	F	White	75+	Knee (x1)	✓		
B6pa	F	White	75+	Knee (x3)		✓	
B7pa	F	White	75+	Knee (x1)	✓		
B8pa	M	White	75+	Both shoulders (more than twice) Hip (x1) Hand (x3) Knee (more than twice) Foot (x2)			✓
B9pa	M	White	45–59	None	-	-	-
C3pa	F	White	75+	Both knees (x5-6)			✓
C4pa	M	White	60–74	Both knees (x6)			✓
C5pa	F	White	75+	Knee(x6) Shoulder (x1) Hand (x1)			✓
C6pa	F	White	75+	Both knees (more than twice)			✓
C7pa	F	White	60–74	Hip (x1) Both shoulders (x1)	✓		
C8pa	M	White	60–74	Knee (x1)	✓		
TOTAL	38 (22 female)		Average 68.5 years Range 49–88 years		9 had single IACs in one or more joints	8 have had multiple IACs in one joint	16 have had multiple IACs in multiple joints

### Clinician participant characteristics

Ten male and nine female clinicians participated including 16 General Practitioners (GPs) and 3 First Contact Physiotherapists (FCPs) from 10 practices. The length of time clinicians had been practicing ranged from 3 to 30 years. The 10 practices represented enabled a sample varied in geographical location and rural and urban environments, serving diverse populations in terms of socioeconomic status, age, and ethnicity. Four clinicians did not administer IACs for osteoarthritis (see Table 2). Interviews lasted an average of 35 minutes (range: 18–49 minutes).

**Table 2:** Clinician participant characteristics.

Participant ID (Practice designated letter, participant number, and professional role i.e., General Practitioner or First Contact Physiotherapist)	Sex (Male / Female)	Years in current role at time of interview	Joints injected for osteoarthritis
A1GP	M	30	Shoulders/knees
I1GP	M	26	Toe/ankle/knee/shoulder
J1FCP	F	6–7	Knee/shoulder
J2GP	F	5–6	Does not currently administer IACS
F2GP	M	6–7	Knees/shoulders
F5GP	M	4–5	Does not administer IACS
H5GP	M	5	Knees/shoulders
H6GP	F	20	Knees/shoulders
G1GP	F	15	Knees/shoulders/wrists
G2GP	F	6–7	Knees and shoulders
E8GP	M	7	Knees
E9GP	M	10–11	Knees
D1FCP	F	10	Shoulders/wrist/thumb/finger joints/knees/ankles
D9GP	F	3	Knees/shoulders
B10FCP	M	6–7	Prescribes but does not administer
B11GP	F	5	Knees/shoulders
B12GP	M	10	Knees
C1GP	M	12	Knees
C2GP	F	5–6	Does not administer IACs
Total	19 (9 Female)	Range 3–30 years	

### Findings from patient interviews

#### Awareness of IACS

Participants reported that IACs were offered by GPs, usually in response to them seeking help for osteoarthritis pain, and mobility difficulties, especially when other treatments (pain medication, physiotherapy) were unsuccessful. IACs were also offered at the point that participants were considering joint replacement surgery, sometimes by an orthopaedic consultant, or a GP to delay/avoid surgery to an affected joint. Participants were positive about receiving IACs during their consultation with the GP. Some patients were referred by their GP to an orthopaedic consultant for their injection. IACs appeared to be commonly offered in this way for advanced osteoarthritis, and patients were generally not aware of it as an option before this. One patient questioned why IACs were not offered more “freely”.

*The surgeon I saw said, ‘Right, we can, um, do you in 15 days’ time, knee replacement’ […] but I thought, ‘Blimey, if you’re going to chop my knee out, I will need to think about this’ [yeah]. And, er, went back to the doctor and he said, ‘Have you never had an injection?’, and I said, ‘No’. He said, ‘Well, I’ll give you one now’, and he gave me the injection and it relieved it. (D5Pa)*

*I think they are absolutely brilliant*, *I always felt quite erm*, *not surprised but… I didn’t understand why they weren’t offered more freely because of how well it worked for me but I guess that’s a personal thing*. *(D3Pa*)

#### Variations in access to IACs

The reason that five patients had not received IACs was that IACs had never been offered, despite some having joint problems for many years before receiving offers of surgery.

*I was a semi-professional [sportsperson] and it was one of these injuries that as a youngster you don’t bother to get seen to, you work through it and the after affects then came on but basically again, I was just allocated pain killers for that. And that was quite over a long period of time. [Yeah, okay, and so, have steroid injections ever been discussed with you?] No, they were never discussed with me, no. (E1Pa*)

The number of IACs patients were allowed within a 12-month period, for each joint affected, varied across the practices. A maximum number of three to four IACs per year, per joint (1 every 3–4 months) was common. Some participants reported that they were permitted only two IACs per year. Some were only offered ‘a one off’ treatment, while others were permitted IACs only once every 2 years. This could be confusing for patients as access often varied depending on which GP they saw in the practice.

*You could only have it done every two years, but I think that’s changed. Then I went back again when it was painful again after two years, I waited and they sent me to have it done again and said I shouldn’t have waited two years, I could have it done whenever I wanted to. So, I think it changes… Every doctor you see, you get a different story. (H2Pa*)

For patients with multiple affected joints, some were given a maximum of two IACs in each knee per year. In an extreme example, one patient was permitted only one IAC every six months, to a single joint, effectively requiring them to ‘choose’ between walking or using their arms.

*I can either have it in the shoulder or in the knee and the knee tends to be painful when I walk and because I’m now not walking, as such, if I want to be able to do anything with my arm—so getting dressed, cooking, cleaning—I’m an artist, a craft-person—I can’t do anything if that shoulder is painful […] the only thing they’re saying is that, you know, ’The steroid is not going to do you any good by having a large amount of it more regularly or in both joints,’ so I have to choose. (D8Pa*)

These experiences indicate considerable variation in access to IACs; and those limitations imposed by GPs on the maximum number of injections can be challenging for those with osteoarthritis in multiple joints. Patients from one practice also reported that accessing IACs became more difficult when more experienced GPs retired. Patients perceived that the new GP was less confident about giving IACs and reluctant to do so for some patients.

*There was two GPs doing it in our surgery and then he retired […] so it was left to another one, who said was going to train up to do it […] to be honest, I didn’t feel that she didn’t seem to know much about just, you know. I know that sounds awful and I’m so sorry for saying it, but she didn’t seem to know a lot and it did hurt […] [Are you going to have any more injections?] They won’t let me. Um, ‘cause the doctor … said she wasn’t keen on it, said [hospital] was going to do it (D4Pa*)

The variation is interesting given that clinical guidelines exist for GPs recommending frequency and criteria for repeat injections [33, 34].

#### Patients’ views of risks and trust

Most patients recalled that GPs or FCPs had discussed the risks associated with IACs with them. Generally, although patients could not describe risks in detail, they had general awareness that “you can’t have them [IACs] too often”. Patients’ recollection of specific risks related to the impact of IACs on joint structure, described as “degeneration of the joint” and “weakening the bone”. Most participants acknowledged but did not express concerns about risks and described trust and confidence in the judgement of the GP.

*Well, I would like to know, is it true that if you have them more frequently you are running a risk of the bone degeneration increasing long term? […] because I do feel very confused. […] So I would really like to know whether frequent steroid injections into the bones will, in the long term, be negative rather than positive, because I’d rather put up with the pain. (H2Pa)*

*There’s this fear that they weaken the bone or something around the joint*, *but I don’t know*. *It’s a balance […] if your joint’s not great anyway*, *you think*, *well*, *you know*, *at least that’ll relieve it for a while [laughs]*. *(D5Pa*)

*I’m very confident in my doctor*. *I don’t think he would have offered it to me if he knew of any bad*, *you know*, *results or… (B1Pa)*

#### The effectiveness of IACs

*Relief of pain and better function*. Patients who had experienced an effective IACs mostly described either complete removal of their joint pain or that pain was less central to their day-to-day experience. Many described that swelling around the affected joint had reduced and some reported a reduction in joint stiffness.

*Up until I had this injection I was walking with a walking stick all the time. But this injection has allowed me to walk without the, I don’t use a walking stick. (B6Pa)*

*Like when it’s near the end I feel like an old bloody man*. *Like getting out the car*, *you get your legs out and then you’re putting pressure on to stand up and that’s when you feel the pain*. *But when it’s done*, *I’m just out the car*, *up and take no notice […] They’re brilliant*. *I love my doctor*, *I said*, *‘God blimey*, *I’m like a new man again now for a few months*.*’ (F4Pa*)

*A return to meaningful activity*. For most participants, relief of osteoarthritis symptoms and restoration of function increased the positive impact on their lives by enabling them to participate in meaningful or important activities, restoring aspects of a ‘normal’ life. By returning to physical activities, patients described being enabled to increase fitness, lose weight and improve comorbidities.

*I was dancing more because my knees weren’t hurting so much […] I would say that certainly the injections enabled me to keep my hobby going. So, there was a considerable benefit. (B4Pa)*

*If I have the injection*, *it does change completely ‘cause it makes*… *My attitude is if I’m out of pain and I can get more healthier by walking (D4Pa)*

*Improvements to mood*. With the reduction of long-term osteoarthritis pain and increased participation in meaningful activities, patients described improvements to mood and emotional health and a restored sense of self. Even patients who did not experience complete pain or symptom relief reported positive benefits in terms of reduced mental burden of pain.

*Well, they’ve made a lot of difference whereas before it was jolly painful, made me… I wouldn’t say a miserable person but made me hard to get going and the pain sometimes you think oh gosh not again, but when I go for the injections again I feel I can get on, I’ve got a totally different outlook on life (C5Pa*)

*Experiences of ineffective IACs*. Some participants reported experiencing ineffective IACs. They attributed this to poor or incorrect placement of the needle into the joint. Two participants who had experienced both ineffective and extremely painful IACs, reported that they believed their IACs had been poorly administered by inexperienced GPs who lacked confidence and training.

*First one was painful, and I could feel the cortisone running down the front of my knee inside my leg so I knew it hadn’t gone in the right place and it never worked at all. [The third IACs] was absolutely perfect […] he presses around my knee and pushes at the soft point where he thinks it’s going to go in the right place, and I somehow know that’s the position. (B6Pa*)

#### Variation in onset and effect duration

Where IACs had ‘worked’ for patients, the interval between administration of IACs and improvement of symptoms varied from immediate to several days or weeks later. Some participants believed the anaesthetic mixed with the IACs was responsible for the immediate effect.

Commonly patients reported that they experienced relief of pain and other symptoms for between two and six months. Some, however, said that benefits lasted a year or more, and some reported that they continued to feel benefit several years after and had not returned to the GP for that particular joint.

Some patients reported that benefits from recent IACs lasted shorter time than their previous IACs. Some attributed this diminishment in duration of effect to worsening of their osteoarthritis.

*The pain relief was instantaneous because they put anaesthetic in it so it kills the pain as it goes in (C8Pa)*

*[How long does it last for*, *the effect*?] *I would say at least three months and you know–yeah I’d say at least three and sometimes a year*. *(C4Pa)*

They don’t help as much as they once did but they do give some relief. (C3Pa)

*I suppose my knees are getting worse*, *so they’re not lasting so long*. *(F4Pa)*

#### An alternative to undesirable treatments

Many participants reflected on how IACs enabled them to avoid more undesirable treatments such as strong ‘painkillers’, or joint replacement surgery. When IACs were effective, they enabled patients to stop or reduce the use of pain relief medicines, due to a superior and more sustained pain-relieving effect. Patients also described a preference for IACs over NSAIDs and opioids, which they said had many unpleasant or debilitating side effects.

*I take Tramadol, I try not to take too much because it can give you a tendency to get mood swings. So, I’d rather put up with a bit of discomfort [having an IACs] than keep filling myself up (B8Pa)*

*If it’s between steroid injections and taking the tablets that upset your whole digestive system then*, *I would go for the injections every time […] It [codeine] just makes you feel immobile because you don’t want to get up and do things*. *(F7Pa*)

Joint replacement surgery is often offered to those for whom other treatments are no longer effective. Many patients had been offered surgery without already receiving IACs, whereas for others surgery was only offered if IACs failed or ceased to be effective. For patients who were unsure about surgery or wished to delay it, IACs were a preferred option if they were effective and available.

*The knee pain was problematic, […*] *my doctor said, ’You need a new knee.’ Like I said, he said that five or six years ago but that hasn’t happened […] that’s how I got to taking the injections. Personally, I think I’ve had a good experience with them, and I wouldn’t be put off taking another one. (H1Pa)*

I mean as I say I wouldn’t contemplate having surgery at my age if I knew I could have an injection and that injection would last me I don’t know, six months. (B1Pa)

*Obviously*, *I’d like to sort of put it off for as long as possible and if doing things like physios*, *exercise and something like these injections sort of keeps you healthy and able to avoid the operation then that’s the approach I prefer to take*. *(B9Pa–not had IACs*)

#### Patient information needs and suggestions for further research

When asked what further information patients would like about IACs or what questions they wanted future research to focus on, many wished to know more about the long-term side effects of IACs on bone health, particularly associated with frequent injection use. The difference between anabolic steroids for muscle growth and IACs was also unclear to some patients. Other suggestions were to give them more frequently at a lower dose to see if it kept pain fluctuations under better control, and to focus research on making IACs with a longer lasting effect.

*The first time I ever heard the word ’steroids’ was at gymnasiums and people that were doing muscle growth and body builders taking steroids. That always looked like a bit of a dirty word for me. (H1Pa)*

*It would be nice to know whether*, *if you’ve had a lot of these steroid injections*, *whether it was safe*, *whether it would make a difference*. *(D2PA)*

*Giving them more regularly*, *maybe a smaller dose*. *So instead of having a big impact*, *that maybe keeping the inflammation under control at some level*, *so that you don’t get these hills and valleys of pain over time*. *(D8Pa)*

#### Findings from interviews with clinicians

Clinicians’ views and beliefs about the IACs are encapsulated by the overarching theme of Caution and Competence. Clinicians felt there is a lack of evidence about the efficacy of IACs and the absolute risks associated with their use. Coupled with their reported lack of engagement with clinical guidance, which they perceived as insufficient, this led to a general sense that clinicians are cautious around the use of IACs. Clinicians also reported a lack of regular training and opportunities to gain experience in administering IACs; insufficient guidance on the technicalities of administering them, and a perception that younger GPs had become more “risk averse” generally in relation to more practical medical procedures. This also led to a sense that their caution is often associated with how competent they felt in administering IACs, given these circumstances. This overarching theme of ‘caution and competence’ can be broken down into a number of subthemes:

#### Confidence and (dis)comfort with practical procedures

Throughout the interviews with clinicians there appeared to be a spectrum of confidence and risk aversion when prescribing IACs, some GPs were more cautious than others. GPs with fewer years in practice were seen by their older more experienced colleagues (and themselves) to be more risk-averse and less confident with the practical administration of IACs. An older, more experienced GP (A1GP) reflected on how previous generations of GPs were more “hands-on” and willing to “give something a try”, whereas current practice involves “more litigation and anxiety surrounding risk”. One GP stated they were not a “*stabby* kind of doctor” much preferring to chat and prescribe medication, which they thought less risky. In contrast one FCP suggested that FCPs who were musculoskeletal physiotherapists were well placed to administer injections because of their specialist knowledge. Confidence and (dis)comfort appear here to be associated with the confidence different clinicians had in accomplishing the procedure competently.

*I’ve done procedural things with injections and scalpels and things, and I found it quite stressful […] I prefer to stick with the medicine side of things. (F5GP does not give injections)*

I don’t dislike doing them but I’m not really a stabby kind of doctor […] I would much prefer to prescribe and chat to someone than start stabbing them because if there’s a problem, you know that’s obviously a lot to sort out. So, they will usually have to persuade me. (C1GP 12 yrs in practice)

*You’ve got to think about it a bit*, *so I find that if you do it*, *they’re incredibly satisfying […] I know a GP who will stick a needle in anything that moves*, *and I know other GPs that won’t go near them*. *I think GPs are becoming more and more risk averse and particularly to practical procedures […] which is a great shame because it provides a good service and a fix for people who otherwise wouldn’t get things done […] and*, *of course*, *we’re brought up now with more litigation and more anxiety surrounding risk*. *(A1GP 30 yrs in practice*)

*Used in the right person at the right time*, *it’s a very effective modality* [*…] It definitely has its place but of course with first contact physios like myself now*, *in primary care who bring the musculoskeletal specialism to primary care*, *you know we’re well placed to do these as well […]*, *it’s all about skill as well*, *something you’re doing time and time again*, *then you maintain your skills*, *and you hopefully become a better injector*. *(D1FCP 10 years injecting joints)*

#### Training

Clinicians’ experience of training appeared to be directly related to their confidence in administering IACs. Training was often provided as an option at various time points during medical training on placements and courses run by a wide variety of providers, or with mentorship from more experienced colleagues. Often only one or two GPs in a practice were trained to provide IACs, which limited availability to patients. More experienced GPs (I1GP) suggested that a lack of confidence and skill amongst newer GPs could be linked to the current training rotation system where trainee doctors are more used to organising referrals or prescribing medicines, rather than doing procedures themselves. Some GPs believed that training should remain ad hoc as they felt it was a relatively easy procedure to learn; while others felt it should be more standardised or even mandated (made compulsory) to ensure quality assurance and education, and to increase its availability to patients.

*I had a very brief orthopaedic job and did some injections then* [*…] So, I just sort of took it up there, it was all kind of ad hoc apprentice based stuff, there was no real formal training […] It’s such a vital skill that it would be nice if the registrars came a little bit more primed ready to start […] I definitely think that it should be more widely available not just particular GPs in particular practices, that isn’t always that helpful. (E9GP)*

*It’s like anything practical as a doctor*, *you kind of go through cycles of confidence doing things*, *and then less confidence*. *But I think I had two or three knees in a row where I really didn’t find it that easy to access the joint space and find myself kind of repositioning the needle or doubting myself a bit […] and think oh goodness me I need a bit more training to be doing this*. *And so*, *I was sort of toying with the idea of not really doing it anymore*. *But since then*, *there’s quite a confident*, *one of our older partners here he’s been doing it for a lot longer*, *he was reassuring and gave me a bit of a hand and taught me some new tips*. *(H5GP)*

*Risk of adverse outcomes*. When asked about the risks of IACs, infection appeared to be of most concern to clinicians. Although they believed the risk was very small. Only two (I1GP, F5GP) mentioned instances when this happened, both of which were during their training. In reference to whether IACs increases the progression of joint degeneration, some clinicians rationalised that the joint was already degrading so progression would be difficult to ascertain, and no GPs or FCPs mentioned seeing any evidence of increased progression. As no clinicians had experienced any major adverse events, they were uncertain of the evidence about the absolute risks of adverse outcomes associated with IACs, and there was a sense that these may be overstated.

*And the risks of infection, I believe are relatively small. Erm, you know side effects are very, very rare. Erm, the risk is that it doesn’t last that long but again if you’re looking at a degenerative joint, even if there is some, you know evidence of increased cartilage wear or you know to the joint, the joint is wearing anyway. (D1FCP)*

*Well to begin with I was always very kind of conscientious in terms of going through the risk of introducing infection into the joint and to present if you had worsening pain*, *a fever*, *swelling or anything like that*. *But we’ve never ever–and we get a lot of injections in this surgery–I’ve never seen anybody who’s had any complications […] Or heard about it*.*”* (*F2GP)*

When discussing risks, clinicians focussed more on adverse outcomes, such as infection or joint degeneration and appeared to separate these from the more general risk of not administering the injection with the correct technique, or a risk that it simply would not provide any benefit.

#### Technical uncertainties

*Needle placement*. There were some technical aspects of the IAC procedure where practice varied between clinicians. Needle placement was often a source of uncertainty for clinicians, with some reporting a lack of confidence when injecting different joints. Confidence with injecting a particular joint varied between clinicians, even for the same joint. Some clinicians thought that success or effectiveness of the injection related to needle placement (A1GP). One FCP suggested that needle placement was not crucial to treatment because the steroid would disperse around the joint capsule.

*You can get less certain at times, particularly if the anatomical landmarks aren’t clear because somebody’s a bit heavier. Particularly shoulders, in fact, are a real issue with that. So yes, not nervous, but just less certain. Reduces your confidence that you’re going to get it in the right place. (I1GP)*

*I think well they say as long as you’re getting the injection into the joint capsule then theoretically the steroid should disperse*, *spread around the joint and give the same effects*. *So as long as you’re getting it into the joint capsule then I think the approach they said doesn’t matter too much*. (*J1FCP)*

*Mixing with local anaesthetic*. Some clinicians used a local anaesthetic mixed in with the steroid, while others did not. Reasons for using anaesthetic was to increase the volume of the injected solution to ensure it fills the joint space. Others used it as a diagnostic indicator to determine that the pain was coming from the joint. If no immediate effect was experienced, it is likely the pain was not attributed to osteoarthritis. Others preferred not to use anaesthetic as they had been told it could leave deposits in the joint itself.

*To be honest I’m not entirely sure how useful the local anaesthetic really is. I think in a way it’s more to sort of bulk out the steroid a little bit so you can wash it out of the vial more effectively. (F2GP)*

*We only use separate [steroid and anaesthetic] in the surgery because that’s historically what we’ve used and I’m not as keen on [steroid brand] because I’ve chatted to surgeons and they say it’s often difficult for them to then go in and pick it out of the tendon because it leaves a residue for a long period of time*. *(E9GP*)

*Variation in frequency of injections*. How often and how many times clinicians administered IACs to a patient varied between clinicians, even in the same practice. Some believed once every 3–4 months was enough, but the age of the patient was a significant factor in judging how many and how often IACs were appropriate. An FCP also suggested no more than three per year, and suggested that when sleep becomes disturbed, indicating an advanced severity, then IACs would be less effective. When considering injecting multiple joints in the same person, one GP (D9GP) suggested that one injection would benefit multiple joints throughout the whole body, which is in opposition to the idea that placement of the needle into the joint capsule is important. Uncertainty is again apparent as they question what their older colleagues might have do in the same situation.

*I just worry a little bit more about repeated steroid into the same joint over a long period of time. I’m not sure what those affects are really, obviously if we do it, it comes with a risk as well, […] infection or damaged tendons or surrounding structures […] But certainly for a fit and young person, you know, with just a bit of degenerative change or pain in a joint, I wouldn’t want to be doing that repeatedly, year on year, multiple times over the year. (E8GP)*

*I would say I would do them*, *maximum*, *every four months*. *If people are older and frailer*, *then I wouldn’t be counting up and saying*, *’You’ve reached 20*.*’ For somebody who is 95*, *I wouldn’t be worried about that at all*. *(D9GP*)

*I tend to say a maximum of three times a year […] And I always try and tease them out a little bit longer so definitely talking about not looking at a calendar but going by symptoms and waiting to that point where normally*, *they can’t sleep*, *so I think once people’s sleep is disturbed then pain becomes a real issue and tiredness*, *and all the things that comes with that*. *(D1FCP)*

*Proximity to surgery*. An area of uncertainty amongst GPs was the issue of timing between an IAC and referral for orthopaedic consultation. We found practice varied between clinicians. While guidance suggests that IACs should not be given within 3 months before surgery, GPs reported that they were informed by local orthopaedic surgeons, some of whom agreed with this guidance. GPs in other localities reported surgeons had suggested that timing made no difference.

*Increasingly I’m injecting people that are on waiting lists for joints* [*…] But now we’re getting to that stage where it’s waiting for so long that I’ll say to a patient, look there’s a small risk that you’re going to get some cancellation and then be told they can’t do an operation right now but the chance of that is so slim I think if you’re in a lot of pain now we can do this injection and give it a go. (H5GP)*

#### Uncertainty about evidence and guidelines

Many clinicians were unfamiliar with the evidence and guidelines for IACs not having reviewed them “for a long time”, most thought that the evidence base was poor, and this influenced their confidence in administering IACs and their confidence in whether it would work or not. Most were certain that if there were any recommended changes to practice, they would hear about them through colleagues, or professional organisations. One FCP referred directly to the NICE guidelines pointing out that they contained insufficient information to guide practice.

*I did a quick internet search myself* [*…] and there isn’t a huge evidence base behind it. I think that informs your confidence in doing a procedure because there’s not a pathway, an established evidence base and when you audit your own work, its 50/50 whether it’s going to work or not […] So yes, it’s very different to other areas of medicine where there are well worn paths and robust evidence, and you feel confident in what you’re doing. (G2GP)*

*NICE guidance just said that it’s a tool that can be used but there is no real guidance*, *as there’s no guidance as to which drug is the best*, *or which dosage is the best*, *you know a lot of the GPs and it seems to be rheumatology as well*, *put big doses in weight bearing joints*, *you know 80mgs*, *and I’ve always been taught to use the*, *well I tend to use 40 erm*, *as per I was taught*, *but I’m not sure if that’s the best dosage to use*. *(D1FCP*)

#### IACs use on the osteoarthritis pathway—“Never my go-to…always an adjunct”

IACs are used at various points along the osteoarthritis pathway although clinicians reported using them most often where core treatments did not work, and as a last resort before surgery. In younger patients, IACs were sometimes used as a diagnostic tool to assess whether the pain was coming from the joint. In those for whom the condition was too early to consider surgery, one FCP used IACs to maintenance of function and continuation of work. Some clinicians used IACs to enable patients to take up other core recommended treatments such as physiotherapy or exercise.

*It’s never my go-to it’s always that adjunct, later on down the line, so I’ll always make sure that within the patient’s history that they’ve at least had attempts at physio and a conservative management. (B10FCP*)

*[Do you ever give it to younger patients*?] *I will happily give a one-off*, *if there’s an acute problem there*, *possibly as part of a diagnostic tool*. *Interested to see if it works*. *I do heavily counsel the patient*, *however*, *this is not a solution to their ongoing problem at that stage (I1GP)*

Some clinicians used IACs as an alternative to other analgesia, seeing fewer options because of changes in the guidelines, but also as an option for patients unable to tolerate medications with particularly debilitating or chronic side effects (drowsiness, mood swings, gastrointestinal problems). One FCP (D1FCP) reasoned that with opioids, older patients were at higher risk of falls, and IACs presented a safer and more tolerable option. Where patients were unfit for surgery because of comorbidities, age or preference, IACs were used more freely by clinicians as they believed no other options were available.

*When you’re weighing up the pros and cons of prescribing things like codeine and titrating up the analgesics, actually the risks of one joint injection if it is lasting, you know at least three months, I think the risks far outweigh the risks of alternative analgesics. (D1FCP*)

Sometimes for elderly people who’ve got, you know, as I say are never going to be fit for surgery and have got OA in multiple joints, I tend to do it maybe three monthly, say four a year. And I might do that as they require really, I’m much more sort of concerned there about, pain relief and functional relief. (E8GP)

#### Perceived benefits and impacts of IACs—“I’m able to just kind of keep them going”

Clinicians often described the effects of IACs as short-term, lasting three months on average with a ‘successful’ injection. There is a sense that this is often seen as a limitation, diminishing the perceived value of IACs. One FCP suggested that this should not be a surprise as corticosteroids only exist in the body for this short period and should not be thought of as a ‘cure’. For some GPs, the use of IACs was ‘incredibly satisfying’ and meant that they could ‘buy time’ and ‘keep patients going’ with good pain relief.

When I’ve got a patient that’s kind of struggling with their pain and they’ve got sort of moderate [osteoarthritis] on the x-ray then sometimes I’m able to just kind of keep them going for a couple of years but they tend to sort of need referral onwards after a couple of years, but I find that it can buy us a bit of time, and get some good pain relief during that time. (B12GP)

*In my experience, some of the injections do incredibly well. And some do very poorly.* […*] you may see an x-ray with advanced changes, but actually, very little inflammatory type pain, so no pain at night, no pain at rest, just weight bearing pain. I find in my experience that those do not do well […] So, I never look at x-rays to decide whether I’m going to inject or not. It’s very much on the clinical picture. And so if somebody’s got active synovitis or a clear inflammatory type pain, so its waking them up at night, can’t sit for too long before stiffening up, then I tend to find the injections do really well […] I tend to find those with night pain do have a good response. And sometimes you know sometimes in the early stage of arthritis, it maybe just that it’s settling a flare and they won’t come back for a long, long time […] I think injections, if they’re used in the right population, will be shown to be very, very effective […] It always surprises me when people say that these injections are short term, well it’s not a surprise because they only exist in the body for up to 12 weeks. And so for a degenerative condition it’s not a cure. (D1FCP)*

#### The possibility of a placebo effect

Some clinicians raised the possibility that IACs elicited a placebo response. Although some felt that any intervention was likely to entail a degree of placebo, others felt that the process of preparing and administering an IAC was particularly powerful. Any potential for placebo effect was not perceived negatively.

*It’s my personal feeling, like any intervention I think like this, you know, you get some benefit, so how much of it is placebo and how much of it is the effect of the steroid it’s difficult to say. (E8GP*)

*A steroid injection feels like a really kind of theatrical event for a GP to do for their patient*. *And you kind of wonder how big a placebo effect and if that kind of has a role in it and then you think well is that a bad thing or not*. *(H5GP)*

#### Clinician information needs and suggestions for further research

When asked what further information clinicians would like about IACs or what questions they wanted future research to focus on, effectiveness of IACs in comparison to other therapies and more evidence on the risks of IACs was suggested. Clinicians wanted more information and better guidance on the longer-term use of IACs, up to five years or more, especially for younger patients, and wanted to know about any risk to surgical outcomes. Clinicians also wanted to know optimal dose and optimal drug; and to see if beneficial effect can be increased or sustained for a longer period with a smaller dose; and whether using saline or anaesthetic to increase volume improved effectiveness. There was also a request for more evidence on the risks of complications between community-based injections and hospital-based injections to ascertain whether more IACs could be given in the community by FCPs, or nurse practitioners to relieve pressures on GP practices. Finally, clinicians also wanted more guidance on the best techniques for administering IACs and better information to give to patients.

*“I guess make sure are there any actual genuine harms that are evident?” (F2GP)*

*“It’s just not something I’ve ever done [giving IACs earlier]*. *But it’s a reasonable thing to do is actually if you do it early and therefore are more able to do physio for which there is reasonably good evidence I believe that it does help*, *then why would we not*? *I don’t know why would we not*. *I guess maybe injecting a younger person*. *Actually no*, *even that why would you not*?*”* (*H6GP)*

“Input from a surgical perspective, just to broaden the base of knowledge that we’ve got, I suppose the long-term implications […] what are the kind of recommendations for say over a three, four, five-year period […] how long can you continue that for before it becomes either a risk or you have to escalate things to surgery?” (B10FCP)

*“I think many more injections could be done in community settings*, *either by physios or by GPs or by nurse practitioners*, *and I think the system benefits are we’re offloading outpatients and patients are getting a more timely treatment*. *(I1GP*)

“Which patients get the most benefit from it” (G1GP)

*It will be a really positive outcome if the outcome were actually the risks have historically been overstated and it has an equal footing with NSAIDS and paracetamol and actually you can do as many as you like*, *you know it doesn’t adversely affect surgical outcomes for example*, *that would be a very interesting and positive outcome for me because it empowers us as*, *you know GPs doesn’t it to get on with and do things*. *(G2GP*)

*I would like to have some clarity on the best techniques for doing them*. *(H5GP*)

#### Areas of convergence

There are a number of converging themes in which the experiences and perspectives of patients and clinicians support and extend one another. Patients seem to become aware of IACs only when clinicians raise the possibility of their use on the osteoarthritis pathway. While some GPs used IACs at various points on the pathway it was usually after other treatments had failed. We can hypothesise that GPs are reticent to mention them as an option before other treatments have been tried, because of a lack of confidence and/or discomfort with administering them. Variation in access to IACs experienced by patients seems to relate in part to the availability of trained clinicians and on the clinicians’ confidence and comfort with administering IACs which stems from uncertainty about the evidence and guidelines, and the more technical aspects such as needle placement and dose and frequency. Patients also thought that the effectiveness of IACs was related to clinicians’ competence and technical factors like needle placement. Patients and clinicians both appeared to question the relative risks of IACs, voicing uncertainty about issues such as accelerated joint degeneration. Patients and clinicians view IACs as relatively low-risk in terms of adverse outcomes, but wanted more evidence on the absolute risks of IACs over long-term and more information and guidance on the optimal number of injections and dosage. Both patients and clinicians valued the use of IACs to reduce symptoms of osteoarthritis and improve patients’ quality of life. Overall confidence in administering IACs however, varied amongst clinicians.

## Discussion

### Other research on experiences and perspectives of IACs for osteoarthritis

A major finding from our study is the variability in access to IACs which is related to GPs cautiousness about IACs and their confidence in being able to accomplish the procedure competently, which in turn is related to ad hoc training and concerns over risks of adverse outcomes and a poor evidence base. Our findings suggest that only a small number of GPs and FCPs within each practice undertake IACs provision. This becomes problematic when a GP leaves a practice and skills are lost, often replaced by more cautious and less competent GPs which further limits patient access. The confidence of these often less experienced GPs is further undermined by the lack of evidence and guidelines for IACs, and clinicians’ knowledge of the guidelines. Furthermore, there appears to be differences in training and how confident clinicians feel at the end of that training, as some appeared to have learnt on the job from an experienced colleague, whereas others had undertaken more structured training. Evidence from UK and international surveys performed between 1992 and 2022indicates that joint injections are mostly performed by small groups of GPs and that confidence and the number of injections they performed was directly linked to their training and competence, while diagnostic uncertainty, and medicolegal concerns are also factors that inhibit use of IACs [35–39]. Our results suggest that little has changed in the past 30 years, and that many GPs are uncomfortable with performing IACs.

There is scant evidence on patients’ and professionals’ views and experience of IACs for osteoarthritis. Two recent studies in America focused on patients’ and clinicians’ decisions about the use of various injection therapies for knee osteoarthritis, including glucocorticoids, hyaluronic acid derivatives, and platelet-rich plasma [40, 41]. The studies found that patients were concerned about the effectiveness, safety, availability and cost of injectable therapies. However these findings are not readily translatable to a UK context as American patients pay all or part of the cost of injections therapies, and the authors do not distinguish between the different injection types [40]. In a study of decision-making for the use of injection therapies, physicians were concerned about the efficacy and risks associated with different injection therapies, the need to manage patient expectations, and the costs [41]. While the transferability of the findings to a UK context is again limited by differences between the US and UK healthcare systems, the authors noted that some physicians were more reliant on clinical guidelines and evidence, while others relied more on their clinical experience, which resonates with findings from this study.

In our study, clinicians and patients were both concerned about the risks of infection and increased joint degeneration. Although both understood these risks to be minimal, clinicians and patients remained unsure of the absolute risks. Current evidence suggests the risk of infection being introduced into the joint (septic arthritis) is very low. A recent study of 22,370 intraarticular injections found only 11 patients were diagnosed with septic arthritis [42]. Clinicians in our study did not inject within 3 months before a joint replacement surgery, and this is supported by current evidence which shows infection after arthroplasty was increased if IACs were given within 3 months but not more than 3 months before surgery [24, 25]. Clinicians’ uncertainty about the risk of increased joint degeneration is reflected in the current evidence in which there is considerable debate [19, 43], with some studies showing an increased risk [20, 22], while others show no deleterious effects [21]. Our study clearly shows that such uncertainty within the evidence base undermines the confidence of clinicians and their decision-making.

In our study not all patients experienced the same beneficial effects of IACs, and the response and duration of response was variable between individuals. While our study is qualitative in nature, these findings are supported by current evidence [15–18]. Some patients referred to long periods of symptom relief, sometimes spanning years. These periods of relief may provide evidence to support the hypothesis that IACs work better for patients with inflammation at the time the IAC is performed, while also justifying what Birrell refers to as the “tear, flare, and repair” model of osteoarthritis, as noted in a recent trial of ultrasound guided IACs for hip osteoarthritis [15, 44]. Clinicians in our study had a propensity to use IACs as a last resort before offering surgery as an option and we can hypothesise that many patients may be towards the end of the inflammatory phase of osteoarthritis when they are offered IACs, therefore cutting short the potential benefit of IACs to serve patients longer as an effective treatment against symptomatic osteoarthritis. Foster et al’s review of osteoarthritis research and treatments highlights the importance of making decisions that match the right patient to the right treatment at the right time [45].

Both patients and clinicians value IACs for osteoarthritis because of their potential to improve quality of life, even for short periods, and as an alternative to other less desirable treatments. These findings provide contextual support for more recent trials, which show that Extended Release IACs can have a “sustained and profound” analgesic effect in people with knee osteoarthritis, sometimes maintained up to 6 months later [6, 46, 47], although these are not yet available in the UK. Our results suggest that patients and clinicians would value IACs that had a longer duration of benefit.

### Recommendations for practice and future research

As the potential demand for IACs increases, we suggest that patients need better access to treatment from clinicians who are more confident and competent in administering them. More regular training and clearer guidelines around the appropriate use and administration of IACs are needed, including optimum interval between repeated IACs, dosage and whether to use local anaesthetic, and better evidence about the absolute risks of infection and impact on cartilage. We also suggest that GPs and FCPs/physiotherapists should be made more aware of the potential use for IACs along all points of musculoskeletal care pathways and that for some patients they can be a preferred and safer alternative to opioids. In discussing our findings with patient representatives, all of whom have osteoarthritis, many had never been offered IACs. Instead, they had only been offered surgery, and were not aware of IAC as an option. We suggest that based on our findings and recent evidence there are not enough GPs with the skills and confidence to offer and administer IACS, which precludes any shared decision-making about their therapeutic use for osteoarthritis. GPs’ confidence with injecting certain joints varied amongst individuals, while the FCP physiotherapist suggested they were better placed to deliver IACs as they had a better knowledge of musculoskeletal anatomy; we therefore suggest that future research should also focus on identifying who is best placed to deliver IACs for osteoarthritis. Additionally, more research is needed to identify their therapeutic use and potential benefits at all points along the musculoskeletal pathway.

### Strengths and limitations

There are no previous studies that we are aware of that have explored patients’ and clinicians’ experiences of using IACs in multiple joints for osteoarthritis. Our data was collected from 10 primary care practices covering diverse populations across the Southwest of England. Combined with an in-depth thematic analysis and a dense, rich data set, this ensured that information power was reached, enabling us to address the research questions in a meaningful way [26]. We chose not to apply a theoretical framework as our intention was to characterise the experiences relevant to the use and administration of IACs within primary care for osteoarthritis. Our pragmatic approach has allowed us to characterise current attitudes of patients and primary care practitioners and enabled us to make recommendations for practice and future research. A common limitation of any research is the self-selecting nature of participation. It was possible that patients who signed up to the study did so because they had positive experiences of IACs. However, as some patients did describe lack of efficacy and limitations of IACs we were able to include a range of experiences. Although the study included some patients and clinicians who had not received or administered IACS, they did not hold negative views of IACs but were simply never offered them in the case of patients, or because GPs colleagues provided them in the practice.

Only one patient described themselves as of “mixed race” while all others identified as “white”. This potentially reduces the diversity of experiences, and while our sampling framework focused on sampling from under-represented populations, practices fed back that ethnicity was not always recorded in patient records. Future research should focus on ways of identifying under-represented groups and should include resource for an interpreter to potentially remove any language barrier. The funding remit of the project was primary care perspectives. Future research could include orthopaedic surgeons’ views to increase understanding of perceived value and risks of IACs for osteoarthritis, as they would be more experienced with the administration of injections and the subsequent treatment of patients who had received IACs. Discussion of our findings with a patient and public involvement (PPI) group assured that our findings were relevant and reflective of wider public experience.

## Conclusion

Our findings suggest access to IACs needs to be improved. IACs are viewed with caution by many GPs, despite being highly valued by patients who benefit from symptomatic relief of osteoarthritis. More evidence about their safety and efficacy is needed from well-designed high-quality trials at all points along the osteoarthritis pathway.

## Supporting information

S1 Appendix Patient topic guide. (PDF)

S2 Appendix Clinician topic guide. (PDF)

## List of abbreviations

NHS: National Health Service
NSAIDs: Non-Steroidal Anti-Inflammatory Drugs
GP: General Practitioners
FCPs: First Contact Physiotherapist
PPIE—: Patient and Public Involvement and Engagement
