# Supplementary material for: Intra-articular corticosteroid injections for osteoarthritis: A qualitative study of patients’ and clinicians’ experiences
Source: PLoS One. 2024 Oct 23;19(10):e0311668. doi: 10.1371/journal.pone.0311668 (PMC11498725; doi:10.1371/journal.pone.0311668)
Supplement: S1 Appendix — (PDF) [file pone.0311668.s001.pdf]

RUBICON-Q Topic Guide - Patients

Introduction: Discuss how the interview will be recorded, issues of confidentiality, anonymisation.
Aim of the study: To understand how people view the use of intra-articular corticosteroid injections, also known as “steroid injections”, for osteoarthritis, their experiences if they have received injections and what they think about future research.
Consent procedure: Check consent to the study
Participant information: Age, employment, ethnic background, practice locale, affected joint and whether received or planning subsequent surgery for arthritis (replacement, fusion, osteotomy, debridement etc), setting in which steroid injection was received if applicable.

Knowledge & views on steroid injections

1. Tell me about your experience of steroid injections for osteoarthritis – have you ever had one, or more injections? IF NOT GO TO Q9.
2. Can you tell me when you had your first steroid injection for OA, and who gave it to you? (GP, physio?)
3. How many appointments did you have to have to receive your injection?
4. How long did you have to wait to get an injection?
5. Can you tell me what effect the injections have had? (e.g. pain relief, increased activity, increased movement)
6. How quickly did you notice any effect and how long did the effects last for?
7. Were there any changes in your day-to-day life after having the injections? Can you tell me about them?
8. Would you have more steroid injections if they were offered, or is this something you would ask about?
9. Why did you decide to have/not have steroid injections?
10. How did you first come to know about steroid injections for osteoarthritis? Was it something *you* raised with your GP or other clinician or was it suggested to you?
11. What names do you know the injections by? (E.g. cortisone, steroid injections etc)
12. What sort of conversations have you had about steroid injections, with any health care practitioner?
13. Were you told that there was a maximum total number of injections or maximum frequency that you could have injections for the joint you had arthritis in?
14. How many GPs in your practice are you aware of that give injections? Are you able to access them easily?
15. What information have you received about steroid injections? Do you feel there is information you would like to know?
16. Are you aware of how steroid injections for osteoarthritis work?
17. Are you aware of any risks involved in having steroid injections? (What are they?)
18. Do you, or did you have any concerns about having steroid injections? (What were your concerns, and did you discuss these with your clinician?)
19. Are you aware of any differences that having a steroid injection, or not, may have had on your future treatment such as surgery? (most surgeons won’t perform joint surgery for between 3-6 mnths after injection due to increased risk of infection)
20. Have you ever had or considered having surgery for your arthritis? Do you feel that steroid injections have or would have made any difference to your decision to have surgery?
21. What difference do you feel steroid injections have made/could make to your quality of life, if any?
22. Do you think steroid injections are an effective way of managing OA?
23. How do you feel about managing your joint pain going forward?

Acceptability of future research & outcomes of interest: – Evidence on the long-term benefits and any risks associated with recurrent use of steroid injections for OA is urgently warranted…

24. As a patient, what do you feel are the most important things to find out about steroid injections for osteoarthritis?
25. Would you potentially be willing to take part in any future research on steroid injections?

Conclusion

26. Is there anything else you would like to add, or anything you wish to talk about that we haven’t covered already?
27. Would you like us to send you a summary of the results of the study once it’s finished?
28. Thank you for participating…END.
