# Supplementary material for: Intra-articular corticosteroid injections for osteoarthritis: A qualitative study of patients’ and clinicians’ experiences
Source: PLoS One. 2024 Oct 23;19(10):e0311668. doi: 10.1371/journal.pone.0311668 (PMC11498725; doi:10.1371/journal.pone.0311668)
Supplement: S2 Appendix — (PDF) [file pone.0311668.s002.pdf]

RUBICON-Q Topic Guide - Clinician

Introduction: Discussion how the interview will be recorded, issues of confidentiality, anonymisation.
Aim of the study: To understand clinicians' perspectives on the use of intra-articular corticosteroid injections for the management of osteoarthritis.
Consent procedure: Check informed consent to the study has been received.
Participant information: Years in practice as a clinician.

- First of all, could you describe your practice for me and the area that you serve?
- Can you tell me about your experience of administering intra-articular corticosteroid injections for osteoarthritis (If you administer them, which joints do you inject?)
- Have you ever had training to administer corticosteroid injections? (What did you think of the training?)
- Can you tell me why you **would / wouldn't** administer corticosteroid injections for OA?
- How do your patients view corticosteroid injections for OAs? (do they ask for them? Are they anxious about them?) - Without telling me anything that I shouldn't know can you tell me about a patient or patients who've had injection/s
- Do you think corticosteroid injections are effective for managing OA from your perspective? What about from a patient perspective?
- Can you tell me what the effects of treatment are for people with OA (e.g. pain relief / increased activity / increased movement)?
- How quickly do patients notice any effect and how long does it last?
- How long would you expect them to work for?
- When do you arrange to review patient's following injection?
- I'm trying to get a feel for how IACIs are administered in different practices – are there other healthcare professionals that have a role in administering corticosteroid injections in your practice? (either administering them or in the decision-making process?)
- Do patients have recurrent injections and if so, what is the limit you use in terms of maximum lifetime number of injections, dosing or maximum frequency of injections?
- Are there any risks involved in administering corticosteroid injections? What risks do you consent patients for? Are there patients who are more high risk?
- Do you have any concerns about giving injections to patients with OA? – What are they?
- What about if the patient is listed for surgery or may be in the future?
- Where do you feel that corticosteroid injections fit best into the osteoarthritis care pathway? (e.g. in relation to joint replacement surgery?)

Evidence base and guidance

- What do you think of the current evidence base for corticosteroid injections for OA? (How have these influenced your beliefs about their use and efficacy?)
- Do you have any views about current guidance on the administering and use of corticosteroid injections for OA?
- There is a concern that as the incidence of osteoarthritis increases, corticosteroid injections will be used more frequently – what are your thoughts on this?

Acceptability of future research including outcomes of interest – robust evidence on the long-term benefits and risks associated with recurrent use of intra-articular corticosteroid injections for osteoarthritis is urgently warranted…

- Would you be willing to take part in any future research on corticosteroid injections?
- What do you feel are the most important research questions we should be asking and outcomes of interest to you as a clinician, and to patients?

Conclusion

- Is there anything else you would like to add, or anything you wish to talk about that we haven't covered already?
- Would you like us to send you a summary of the results of the study once it's finished?
- Thank you for participating…END.
